# Results of Lower Limb Bone Lengthening by Using Motorized and Magnet-Driven Intramedullary Nails to Treat Limb Length Discrepancy

**DOI:** 10.7759/cureus.77212

**Published:** 2025-01-10

**Authors:** Mohammed J AL-Sayyad, Fahad A Alshoaibi, Ammar A Alshuaibi, Ayman F Almohammadi, Hatim A Almaghrabi, Abdullah M Alsaady, Jalal M Alsayyad

**Affiliations:** 1 Department of Orthopedics, King Abdulaziz University Hospital, Jeddah, SAU; 2 Faculty of Medicine, King Abdulaziz University, Jeddah, SAU

**Keywords:** complication, fitbone, intramedullary nails, limb length discrepancies, precice

## Abstract

Introduction: Limb length discrepancies (LLDs) can be treated with limb lengthening using external fixation or intramedullary nails, such as PRECICE or FITBONE. PRECICE is a magnetically driven titanium nail, while FITBONE is a motorized nail; both use an external remote control. This study aimed to determine the complications, compensation of length difference, time needed for compensation, and healing outcome of bone lengthening using motorized or magnet-driven intramedullary nails among non-cosmetic limb lengthening cases.

Methods: This retrospective study was derived from a single surgeon’s practice at a tertiary hospital in Jeddah, Saudi Arabia. Between 2009 and 2020, 15 patients with LLDs, with a mean age of 25.43 years (range 16-35 years), were identified. The study included congenital, post-traumatic, idiopathic, and acquired causes of LLDs. Outcomes were measured using complete lengthening and healing indices, which need the presence of three intact cortices out of four.

Results: All patients achieved correction of their limb length without any device failure or nail breakage. The achieved limb length for all patients averaged 38.4 mm, ranging from 21 to 60 mm. No intraoperative complications were observed. Postoperatively, one patient reported knee stiffness, and another developed deep venous thrombosis. Duration of distraction (average 43.93 days, range 30-66 days) and the mean healing index was 32 days/cm for both the femur and tibia.

Conclusion: This study confirms that intramedullary nail PRECICE 2 and FITBONE systems are reliable and effective lengthening devices for treating lower LLDs and deformities. These devices significantly reduce complications and achieve excellent outcomes.

## Introduction

Limb length discrepancies (LLDs) can have traumatic or congenital causes [1,2]. LLDs can lead to secondary degenerative changes, back pain, and limping [3]. Bone lengthening can treat this problem either by external fixation methods or intramedullary nails, such as the PRECICE 2 nail ® (Nuvasive, INC, Sandiego, CA, USA) and FITBONE ® (Orthofix, Lewisville, TX, USA) systems [1,4,5]. External fixators have traditionally been used in distraction osteogenesis, but they have been associated with several problems, such as pin site infection, joint stiffness, muscle contracture, malalignment, and re-fracture.

In recent years, implantable devices have become increasingly common [1]. PRECICE, a magnetically driven titanium nail, is one of the most common lengthening nails. By contrast, the FITBONE system uses a motorized nail. Both systems use external remote controls to ensure precise and consistent lengthening control [6,7]. Intramedullary lengthening devices have been introduced over the past two decades to reduce overall complication rates and improve patient satisfaction [6-13]. Compared with external devices, intramedullary lengthening nails provide several benefits in the management of LLD, including reduced rates of infection, less pain and soft tissue damage, better joint movement, and enhanced alignment control [13].

Since Bliskunov invented the first telescopic nail in 1983, our understanding of bone lengthening has significantly advanced, leading to considerable technological advancements and the creation of motorized, externally controlled lengthening devices [4,14]. One in 2000 children is thought to have significant lower limb malformations and limb length differences (>2 cm) and will likely benefit from these techniques [10].

Lower limb lengthening with fully implantable intramedullary nails is a substitute technique thought to lower complications and increase patient satisfaction. The PRECICE and FITBONE intramedullary limb lengthening systems provide accurate distraction using an external remote control with great success and safety [11].

Only one previous report from Saudi Arabia on the use of these nails to lengthen bones was found on our literature review, which was written by the senior author of this paper [7]. In contrast to this previous research, which included cosmetic cases, our study excluded patients undergoing intramedullary lengthening for cosmetic purposes. Our aim was to focus exclusively on patients who underwent the implementation of PRECICE or FITBONE systems for lower LLDs.

This study aimed to determine the complications, compensation of length difference, the time needed for compensation, and healing outcome of bone lengthening using motorized and magnet-driven intramedullary nails to treat LLD among patients with congenital LLD, post-traumatic LLD, idiopathic LLD, and other acquired LLDs at a single tertiary health center in Jeddah, Saudi Arabia.

## Materials and methods

Study design

This retrospective database study was derived from a single surgeon’s practice between 2009 and 2020 at a single tertiary healthcare center at King Abdul-Aziz University Hospital (KAUH), Jeddah, Saudi Arabia. The KAUH Ethics Committee approved this study (reference number: 456-22). Data were extracted and prepared using Microsoft Excel (Microsoft Corporation, Redmond, WA, USA). 

Patients were selected based on the following inclusion criteria: congenital, acquired, and post-traumatic LLDs that were lengthened using intramedullary lengthening nails with a minimum of two years of follow-up. Patients who had undergone intramedullary lengthening for cosmetic purposes were excluded.

To achieve lengthening, we used two systems: the FITBONE Telescope Active Actuator, a completely implantable, motorized intramedullary nail that comes in different sizes and designs for both the femur and tibia [15], or the PRECICE nail, which is a fully implantable magnetic-controlled expansion system.

Patient demographic data included medical record number, age, sex, demographic area, body mass index, smoking status, medical comorbidities, chronic diseases, type of nail used in the surgery, etiology, complications, length difference, desired length, achieved length, type of deformity, and previous surgical history.

Preoperatively, all patients underwent clinical and radiographic measurements using X-ray and CT scanograms to evaluate their leg length discrepancy. The implantation technique for the PRECICE nail was performed by Paley [16]. The technical details of the FITBONE nail insertion can be found in another article [7]. Two 3.0 mm Kirschner wires were used to control the rotational alignment in the proximal and distal segments. The osteotomy technique used in this procedure was drill-hole corticotomy. Straight reamers were employed to enlarge the medullary canal by 0.5 mm greater than the diaphyseal diameter of the nail. After nail insertion, in cases where the FITBONE system was used, a subcutaneous reception antenna was connected to the motor in the proximal part of the nail through a thin, insulated, flexible wire. Baumgart’s reverse planning method was used in cases where deformity correction was planned [17]. Blocking screws were used to maintain deformity correction throughout the procedure [7]. In both nail systems, the nails were tested for function after the completion of locking.

Following the surgical procedure, all patients received a daily dose of 40 mg enoxaparin sodium postoperatively for prophylactic purposes with a six-day resting period, followed by distraction therapy. For femoral lengthening, three sessions per day were conducted, producing a distraction rate of 1 mm/day, whereas for tibial cases, two sessions per day were conducted, producing a distraction rate of 0.67 mm. Weekly clinical follow-ups were conducted during the distraction phase, with additional follow-ups after three, six, nine, 12, and 18 months to record the range of motion of the hip, knee, and ankles and any issues or complications. Patients underwent daily physiotherapy programs, including stretching and strengthening around the knee and ankle joints. During the distraction phase, radiographs were taken every week, while during the consolidation phase, they were taken every four weeks. Anteroposterior and lateral radiographs were taken to determine consolidation once the distraction gap was closed on three sides, and the patient was able to stand without crutches [4].

Primary outcome measures included a completed lengthening and consolidation index, which was calculated as the ratio of the duration required for bone healing and the achieved length (day/cm).

Statistical analysis

Data were gathered and organized using Microsoft Excel (Microsoft Corporation, Redmond, WA, USA). Data were expressed as mean +/- standard deviation (minimal - maximum) for parametric data and frequency (%) for categorized data. Analysis was made by IBM SPSS Statistics for Windows, Version 23.0 (released 2015, IBM Corp., Armonk, NY). Shapiro-Wilk test was employed to determine the normality of parametric value distributions. Wilcoxon test was utilized for pre- and postoperative mMTPA and mLDFA as parametric data were abnormally distributed and the Kruskal-Wallis test was for the comparison of length difference, length archived, duration of distraction, and length of hospital stay due to the small sample size. Fisher's exact test was used for the comparison of categorized data (types of nails) as appropriate. P < 0.050 was considered statistically significant.

## Results

Fifteen patients with LLD were involved in the study, comprising nine males (60.0%) and six females (40.0%) with a mean age of 25.43 years (range 16-35 years) and mean BMI of 27 kg/m^2^ (range 23-40 kg/m^2^). One patient (6.7%) was smoking. The causes of LLD included traumatic injury in five (33.3%), congenital causes in five (33.3%), poliomyelitis in one (6.7%), Perthes disease in one (6.7%), cerebral palsy with iatrogenic LLD in one (6.7%), and idiopathic causes in two patients (13.3%). Two patients (13.3%) had genu varum deformity (13°, 26°), one (6.7%) had genu valgum (4°), and one (6.7%) had coxa vara (120°), as shown in Table 1.

**Table 1:** Demographics and etiology. Data are expressed as mean +/- standard deviation (minimal – maximum) for parametric data and frequency (%) for categorized data.

Data	Value
Age (years)	25.47 ± 6.41 (16-35)
BMI (kg/m^2^)	27.02 ± 4.37 (23-40)
Gender	
Male	9 (60.0%)
Female	6 (40.0%)
Smoking	1 (6.7%)
Etiology	
Congenital	5 (33.3%)
Trauma	5 (33.3%)
Idiopathic	2 (13.3%)
AVN*	1 (6.7%)
Polio	1 (6.7%)
Iatrogenic	1 (6.7%)
Deformities	
No	11 (73.4%)
Genu varum (13°, 26°)	2 (13.3%)
Genu valgum (4°)	1 (6.7%)
Coxa vara (120°)	1 (6.7%)

Table 2 shows that the femur was the segment involved in 13 patients (86.7%), while the remaining two (13.3%) underwent tibial lengthening. For all the patients, the mean length difference was 38.43 mm (range 22-60 mm), the mean length achieved was 38.40 mm (range 21-60 mm), mean duration of distraction was 43.93 days (range 30-66 days), and mean duration of hospital stay was 3.60 days (range three to five days). FITBONE was mostly used in 13 patients (86.7%); of them, 11 were used with the femur and two were used with the tibia. Meanwhile, PRECICE nails were used in two (13.3%) with a significant difference between groups (p = 0.005). The mean preoperative leg length discrepancy for the femoral bone with the FITBONE nail was 35.73 mm (range 22-60 mm); for the femoral bone with the PRECICE nail, it was 52.25 mm (range 44.5-60 mm); and for the tibia bone with the FITBONE nail, it was 39.50 mm (range 29-50 mm), with insignificant difference between groups (p = 0.313). The mean length achieved for the femoral bone with the FITBONE nail was 35.73 mm (range 21-60 mm); for the femoral bone with the PRECICE nail, it was 52.50 mm (range 45-60 mm); and for the tibia bone with the FITBONE nail, it was 39.0 mm (range 29-49 mm) with insignificant difference between groups (p = 0.311). The mean duration of distraction for the femoral bone with the FITBONE nail was 41.36 days (range 30-66 days); for the femoral bone with the PRECICE nail, it was 57.5 days (range 50-65 days); and for the tibia bone with the FITBONE nail, it was 44.50 days (range 34-55 days), with insignificant difference between groups (p = 0.379). The mean duration of hospital stay for femoral bone with the FITBONE nail was 3.55 days (range three to five days); for the femoral bone with the PRECICE nail, it was 3.50 days (range three to four days); and for the tibia bone with the FITBONE nail, it was four days, with insignificant difference between groups (p = 0.522).

**Table 2:** Results of the bone involved and nails. Data are expressed as mean +/- standard deviation (minimal – maximum) for parametric data and frequency (%) for categorized data. The Kruskal-Wallis test was used for the comparison of length difference, length archived, duration of distraction, and length of hospital stay due to the small sample size, and Fisher's exact test was used for comparison of categorized data (types of nails).

Items	All patients (n = 15)	Femur with FITBONE (n = 11)	Femur with PRECICE (n = 2)	Tibia with FITBONE (n = 2)	Significance
Type of nail					P = 0.005
FITBONE	13 (86.7%)	FITBONE (100%)	-	FITBONE (100%)	
PRECICE	2 (13.3%)	-	PRECICE (100%)	-	
Length difference (mm)	38.43 ± 12.64 (22-60)	35.73 ± 11.96 (22-60)	52.25 ± 10.96 (44.5-60)	39.50 ± 14.85 (29-50)	P = 0.313
Length achieved (mm)	38.40 ± 12.56 (21-60)	35.73 ± 11.91 (21-60)	52.50 ± 10.96 (45-60)	39.0 ± 14.14 (29-49)	P = 0.311
Duration of distraction (days)	43.93 ± 12.51 (30-66)	41.36 ± 11.90 (30-66)	57.50 ± 10.61 (50-65)	44.50 ± 14.85 (34-55)	P = 0.379
Duration of hospital stay (days)	3.60 ± 0.63 (3-5)	3.55 ± 0.68 (3-5)	3.50 ± 0.71 (3-4)	4.00 ± 0.00 (4-4)	P = 0.522

The mean healing index was 32 days/cm for both the femora and tibiae. There was no statistically significant difference between the tibiae and femora. Similarly, no statistically significant differences existed between patients with and without preoperative deformities.

Table 3 reveals that there were insignificant differences between the pre- and postoperative mMTPA (p = 1.00) and mLDFA (p = 0.750). Moreover, there were insignificant differences between the pre- and postoperative mMTPA and mLDFA in patients with femur with FITBONE (p = 1.00 and p = 0.062), femur with PRECICE (p = 1.00 and p = 0.317), and tibia with FITBONE (p = 1.00 and p = 1.00).

**Table 3:** Comparison of the pre- and postoperative angles in different groups of patients. Data are expressed as mean +/- standard deviation (minimal – maximum). Wilcoxon test is utilized for the pre- and postoperative mMTPA and mLDFA as parametric data were abnormally distributed.

Items	All patients (n = 15)	Femur with FITBONE (n = 11)	Femur with PRECICE (n = 2)	Tibia with FITBONE (n = 2)
Pre mMTPA	87.00 ± 0.00 (87-87)	87.00 ± 0.00 (87-87)	87.00 ± 0.00 (87-87)	87.00 ± 0.00 (87-87)
Post mMTPA	87.00 ± 0.00 (87-87)	87.00 ± 0.00 (87-87)	87.00 ± 0.00 (87-87)	87.00 ± 0.00 (87-87)
Significance	p = 1.00	p = 1.00	p = 1.00	p = 1.00
Pre mLDFA	88.57 ± 8.90 (80-113)	90.20 ± 9.24 (83-113)	82.00 ± 2.83 (80-84)	87.00 ± 0.00 (87-87)
Post mLDFA	86.57 ± 2.027 (80-89)	87.20 ± 0.79 (86-89)	83.00 ± 4.24 (80-86)	87.00 ± 0.00 (87-87)
Significance	p = 0.750	p = 0.625	p = 0.317	p = 1.00

**Figure 1:**
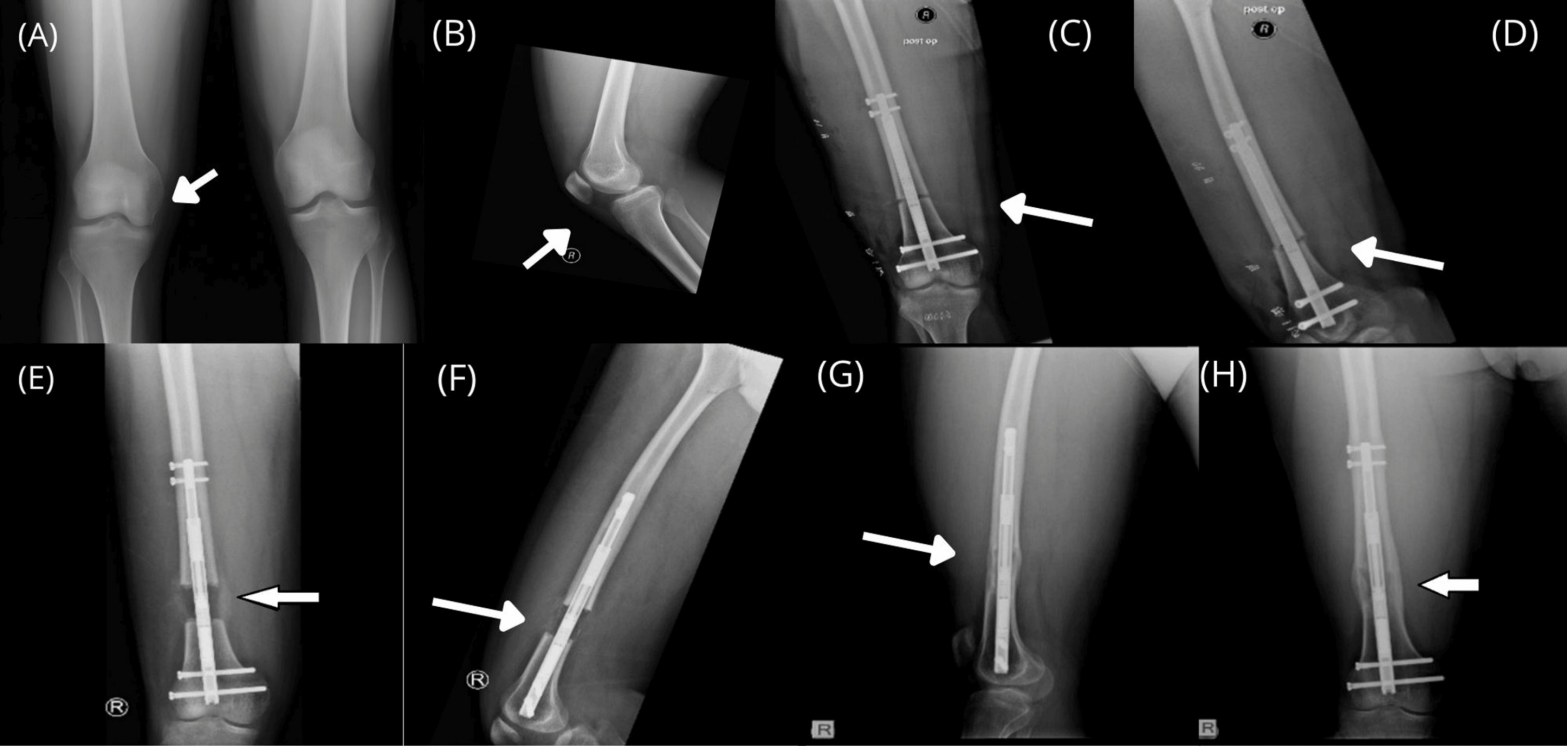
Radiographs Radiographs of a 24-year-old patient. A) Preoperative bilateral AP view, B) preoperative lateral view, C) immediate postoperative AP view, D) immediate postoperative lateral view, E) during lengthening AP view, F) during lengthening lateral view, G) AP view after healing of the lengthening site, H) lateral view after healing of the lengthening site.

Figure 1 shows some radiographs of our patient who used the PRECICE nail. 

No intraoperative complications were identified. No infections of the bones or soft tissues were observed. One patient developed knee pain and stiffness, necessitating more extensive physiotherapy, which required a slowing down of the distraction rate and physiotherapy. After completion of distraction, we removed the FITBONE antenna because of knee pain, and the stiffness was resolved. Another patient with poliomyelitis developed deep venous thrombosis. Following the course of anticoagulation, the patient’s condition resolved without any further issues.

## Discussion

Lengthening using intramedullary limb lengthening nails is popular because of documented complication degrees compared to external fixation use, with a high satisfaction rate [4,18,19]. 

This retrospective study analyzed 15 patients with congenital, post-traumatic, idiopathic, and acquired LLDs in a single tertiary health center in Jeddah, Saudi Arabia. We assessed the joint complications, compensation of length difference, time required for compensation, and healing outcome of bone lengthening by using motorized and magnet-driven intramedullary nails to treat LLDs.

A study conducted by the same author of this paper involved a case series with 10 patients who performed lengthening with the FITBONE intramedullary nail. The findings revealed an average consolidation index of 24 days/cm (range 20-39 days/cm). Patients achieved their desired length with an average of 96.6%. The FITBONE approach showed several advantages compared to the external fixation method, including a lower risk of infection, early full weight-bearing, and accelerated rehabilitation. In addition, it allowed for easier motor control and better postoperative alignment of the mechanical axis. However, this series involves two cosmetic lengthening [7].

Several studies have indicated that the PRECICE nails and FITBONE nail systems are effective and accurate for lengthening. In a retrospective study of 26 implanted PRECISE nails, the average attained length was 37 mm (15-50 mm) [20]. Kirane YM et al. conducted a similar study involving 24 individuals and found that the average overall length was 35 mm (14-65 mm) [21]. Singh S et al., a retrospective investigation of 24 implanted FITBONES, revealed that the average length achieved was 40 mm (27-60 mm) [15]. Our findings support previous evidence of the efficiency of both FITBONE and PRECICE nails; the average length attained was 38.4 mm (21-60 mm).

Our study showed similar results to the previous repeated lengthening nail reliability and consolidation index with a low rate of complication [18,22-24].

Various categorization approaches for problems in lengthening operations have been published, some of which occurred before the widespread availability of internal lengthening [25,26]. The mechanical difficulties associated with implanted nails can be split into two categories: failure of the distraction mechanism and the nail's integrity breakage. No instances of failure of the lengthening mechanism were observed in this series. On the other hand, joint stiffness is a common problem in limb lengthening. Joints are reportedly the second most common source of complications, which is consistent with our results [27,28]. According to Teulières et al., 5.8% of 34 patients had postoperative problems that impacted long-term outcomes, which was not the case with our findings [3]. We monitored one patient with knee stiffness, reduced the distraction rate, and initiated an intensive physiotherapy regimen [29]. Iliadis et al. reported a patient without comorbidities who developed deep venous thrombosis [4]. In our series, one patient with poliomyelitis developed deep venous thrombosis despite being on anticoagulants during the lengthening period. The patient was treated medically with no long-term complications despite being on anticoagulants during the lengthening period.

This study has some limitations that should be considered. The sample size was small, and there was no objective assessment of functional outcomes. Given these limitations, conducting a follow-up study to address these issues would be beneficial.

## Conclusions

This study demonstrated successful bone lengthening using either FITBONE motorized nails or PRECICE intramedullary nails for the femur and tibia in individuals with inherited, acquired, or post-traumatic conditions. Nevertheless, intramedullary lengthening is associated with risks and complications, including deep venous thrombosis and knee stiffness. Complex deformities can be corrected with a low complication rate, meticulous preoperative planning, and accurate surgical techniques.
